# Hemorrhagic Fever with Renal Syndrome: Pathogenesis and Clinical Picture

**DOI:** 10.3389/fcimb.2016.00001

**Published:** 2016-02-03

**Authors:** Hong Jiang, Hong Du, Li M. Wang, Ping Z. Wang, Xue F. Bai

**Affiliations:** ^1^Center for Infectious Diseases, Tangdu Hospital, Fourth Military Medical UniversityXi'an, China; ^2^Department of Microbiology, School of Basic Medicine, Fourth Military Medical UniversityXi'an, China

**Keywords:** hantavirus, hemorrhagic fever with renal syndrome, *Bunyavirus*, hantaan virus, pathogenesis

## Abstract

Hantaan virus (HTNV) causes hemorrhagic fever with renal syndrome (HFRS), which is a zoonosis endemic in eastern Asia, especially in China. The reservoir host of HTNV is field mouse (*Apodemus agraricus*). The main manifestation of HFRS, including acute kidney injury, increases vascular permeability, and coagulation abnormalities. In this paper, we review the current knowledge of the pathogenesis of HFRS including virus factor, immunity factor and host genetic factors. Furthermore, the treatment and prevention will be discussed.

## Introduction

Hemorrhagic fever with renal syndrome (HFRS) has been a major epidemic mainly in Asia and Europe. About 100,000 cases of HFRS are documented annually (Zhang et al., 2008), most of which occurred in China, Korea, and Russia (Yu et al., 2014). In northern Europe, mostly in Sweden and Finland, the case-fatality rate of nephropathis epidemica (NE) was 0.1–1% (Hjertqvist et al., 2010). SEOV causes HFRS with a mortality rate of 1% (Saksida et al., 2011). Among all the countries, China is the most seriously affected one which accounts for over 90% of the total HFRS cases around the world (Khaiboullina et al., 2005a; Jonsson et al., 2010). During 1950–2014, a total of 1,625,002 cases and 46,968 deaths were reported in China, with the death rate of 2.89%, according to the statistical data from the national health and Family Planning Commission of China.

HFRS is caused by Hantaan virus (HTNV), Amur virus(AMV), Seoul virus (SEOV), Dobrava virus (DOBV), or Puumala virus (PUUV), each of which causes diseases with differing severity. HTNV or DOBV may cause the most severe form of HFRS and have the highest morbidity rates ranging from 5 to 10% (Jonsson et al., 2010) SEOV is globally widespread and may cause moderate HFRS; PUUV is endemic in northern Europe and may cause a generally mild form of HFRS, which is also called NE. Sin Nombre virus (SNV), which belongs to the same genus as the former ones which is Hantavirus, is associated with a severe form of hantavirus cardiopulmonary syndrome (HCPS).

Hantavirus mainly infects human vascular endothelial cells and causes extensive damage in capillaries and small vessels. However, the explanation of the pathogenesis of HFRS is far from enough. One of the reasons is the lack of suitable animal models. Recently, many basic and clinical studies are shedding light on its underlying mechanisms and suggesting various means to reduce the severity of illness. In this paper, we review investigations of the pathogenesis of HFRS and speculate on directions for future studies. We will first introduce basic information of hantaviruses, and then the rodent reservoirs and routes of transmission to humans. After that, we will summarize the clinical features of HFRS and review the major pathogenesis, mainly focusing on the role of innate and adaptive immune responses in producing vascular leakage, coagulation defects and other features of the disease. Finally, we will discuss the recent advances in treatment, and focus on some new treatments that could reduce deleterious host responses to infection.

## Hantaviruses and their reservoir hosts

HTNV is an enveloped virus, with a negative-sense RNA genome, which belongs to the *Hantavirus genus* within the family of *Bunyaviridae* (Kaiwa et al., 2007). The virus particle is oval or spherical in shape with a diameter ranging from 80 to 120 nm. The genome of HTNV-RNA consists of three segments designated S [small: 1700–2100 nucleotides(nt)], M(medium: 3600–3700 nt), and L(large: ~6500 nt). The S segment encodes the nucleocapsid protein; the M segment encodes envelope glycoproteins (G1 and G2); L segment encodes the RNA-dependent polymerase protein (Jiang et al., 2008; Hall et al., 2010). HTNV is very stable and can remain infective for 2 weeks at room temperature and presumably for more time at lower temperature.

HTNV and SEOV are two serotypes in China, with the main natural hosts being *Apodemus agrarius* and *Rattus norvegicus*, respectively (Zhang et al., 2014a). These reservoir animals are asymptomatic after infection because the hantaviruses have developed different escape mechanisms against the innate immune system during the long lasting co-evolution within these different hosts species (Rang, 2010).

The main transmission pathway from rodents to humans is aerosolized excreta inhalation and contact infection. Person-to-person transmission has not been found. Rural areas, with poor housing conditions and high rodent density, accounts for more than 70% of HFRS cases, and the majority of infected cases are local farmers (Zhang et al., 2010). Forest workers, shepherds, woodcutters and military personnel also have high occupational hazard from HTNV infection in that epidemiologic investigations have linked virus exposure to such activities as heavy farm work, threshing, sleeping on the ground and military exercises (Schmaljohn and Hjelle, 1997).

HFRS mostly occurs in the period from the late autumn to the next spring, with two incidence peaks (Liu et al., 2007). The starting time of HFRS incidence peak depends on the type of pathogenic hantavirus and also coincides with increased human outdoor activities in spring and fall. The scale of rodent populations may also affect the disease outbreaks in humans (Khaiboullina et al., 2005a). SEOV is the major hantavirus that circulates in the domestic rats and causes human infection in urban areas. SEOV-caused HFRS mainly occurs in spring.

## General features of hemorrhagic fever with renal syndrome

The clinical course of HFRS is primarily characterized by fever, circulatory collapse with hypotension, hemorrhage, and acute kidney injury (AKI). The disease typically progresses through five phases: febrile, hypotensive shock, oliguric, polyuric, and convalescent (Table 1). Additionally, some of these phases frequently overlap in severe cases, and one or two phases are frequently absent in some mild cases. Laboratory findings during acute stage of the disease are anemia, leukocytosis, thrombocytopenia, elevated liver enzymes, and serum creatinine (renal dysfunction), as well as proteinuria and hematuria. Most of the cases can recover completely, while some severe cases still have some sequelaes including headache, insomnia, hyperhidrosis, hemorrhage, and hyperdiuresis.

**Table 1:** **Typical clinical phases, symptoms and complications of HFRS**.

**Phase of illness**	**Febrile**	**Hypotensive**	**Oliguric**	**Polyuric**	**Covalescent**
Time of occurence	1~7 days	1~3 days	2~6 days	2 weeks	3~6 months
Principal features	Fever	Hypotension	Urine output decreased	Urine output increased	
Signs and symptoms	Headache	Capillary leakage	Anuria (urine output < 100 ml/day)	Weight decrease	Weakness
	Vomiting	Pulmonary symptoms	Fluid retention		Fatigue
	Abdominal pains				
	Back pains				
	Visual disturbances				
Complications	Acute encephalomyelitis, Bleedings, Multiorgan failure, Pituitary hemorrhage, Glomerulonephritis, Pulmonary edema, Shock, ARDS, DIC				

Kidney injury frequently occurs in HFRS and the most prominent pathological presentation is acute tubulointerstitial nephritis following the infiltration of inflammatory cells (Kim et al., 2010). AKI often induces death in patients with HFRS, particularly in the oliguric phase (Wang et al., 2013). The elderly patients often develop severe AKI and are more likely to have shock, hematuria, thrombocytopenia and leukocytosis. The patients with severe AKI usually need dialysis or continuous blood purification and stay longer in hospital than non-AKI patients (Wang et al., 2013).

Thrombocytopenia, which is one of the factors that cause the increase of blood vessel permeability, is related to severe AKI among patients with acute HTNV infection. Acute thrombocytopenia is a common symptom of HFRS and persists throughout hantavirus infection. Therefore, thrombocytopenia is an important basis for the diagnosis of HFRS (Denecke et al., 2010).

Pulmonary, cardiac, endocrinological, central nervous system, and ocular findings are also major manifestations of HFRS. The febrile, hypotensive, and oliguric phases can overlap in some severe cases. In this condition, acute progressive noncardiogenic pulmonary edema, which often presents as acute respiratory distress syndrome (ARDS), is likely to happen, and, thus, results in a high fatality rate. It was demonstrated that the agitation, conjunctival hemorrhage, coma, were also negatively correlated with survival outcome (Du et al., 2014a).

The diagnosis of HFRS is based on exposure history, typical clinical manifestations, and serum test results, such as the detection of IgM or IgG antibodies against hantavirus in patient serum by enzyme-linked immunosorbent assay (ELISA) and colloidal gold method. A safe, rapid and specific serotyping method for diagnosis was developed by using the recombinant hantavirus nucleocapsid protein (NP) as antigen (Li et al., 2006). It has been demonstrated that the detection of NP-specific IgM antibodies in clinical samples is a good indicator of a recent hantavirus infection (Peters and Khan, 2002). The detection of hantavirus-specific IgM in an ELISA format is the most valuable and widely used method for diagnosing acute hantavirus infections (Li et al., 2002). Truncated recombinant NP detection has been shown to be more specific to differentiate the involved hantavirus serotype (Araki et al., 2001). However, ELISA and other serological tests cannot be used to assess the replication of the virus in patient's blood. Several methods for detection of HTNV in cell culture supernatant or blood sample have also been developed. Plaque assay is a classical method to titrate virus. However, it usually takes 5–7 days for the formation of the viral plaques. Recently, a SYBR Green I based real-time PCR assay targeting the S segment of the HTNV genome for the detection of virus in cell supernatant was developed (Jiang et al., 2014).

## Pathogenesis of HFRS

**Figure 1:**
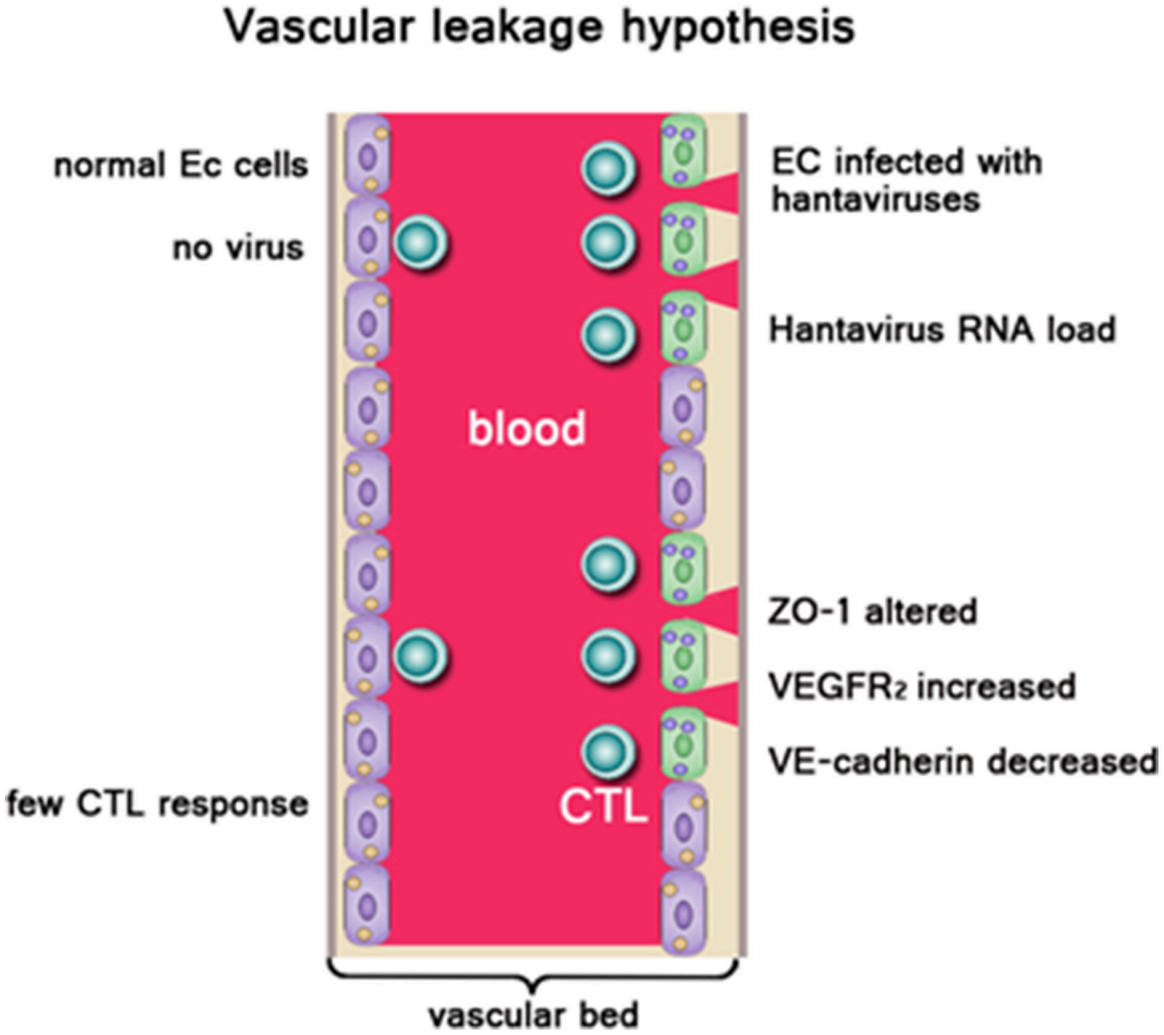
**Left side:** Normal endothelial cells (EC), no vascular leakage occurs. **Right side:** EC were infected with hantaviruses. ZO-1, VEGFR2, VE-cadherin on EC were altered. High hantavirus RNA load result in severe vascular leakage. Virus-infected ECs be cleared by virus-specific CTLs leading to vascular damage. Owing to acute thrombocytopenia, there are not sufficient platelets available to repair “holes” in the EC barrier, resulting in vascular leakage. In addition, cytokines produced during the innate response against pathogenic hantaviruses like TNF-α could enhance vascular permeability.

HFRS has been widely believed as one type of systemic inflammatory response syndrome, and the patient's pathophysiologic manifestations of the hypotensive phase are similar to those of typical distributive shock. Vascular endothelial dysfunction is the basic pathological change, which is characterized by dramatic increase in vascular permeability (Grvrilovskaya et al., 2008; Gorbunova et al., 2011). Increased vascular permeability leads to plasma exosmosis and even hemorrhage, which is associated with lots of clinical features in HFRS, such as hemoconcentration, hypotension, shock, and abdominal pains (Hayasaka et al., 2007) (Figure 1). So far, it is difficult to explain the pathogenesis in a single factor. Studies have suggested that HFRS may be developed via indirect mechanisms including the virus factors, immune factors and host genetic factors.

### Virus factor

HFRS is characterized by increased vascular permeability and coagulation disorders. It was recognized that human endothelial cells isolated from both adult and fetal veins are highly susceptible to the HTNV infection. However, *in vitro* infection with HTNV does not cause any noticeable cytopathic effect, as judged by both phase microscopy and electron microscopy (Pensiero et al., 1992). Therefore, hantavirus is considered to be a non-cytopathogenic virus which targets primarily vascular endothelial cells (Guhl et al., 2010).

It has been demonstrated that there is an association between the hantavirus RNA load and disease severity in some recent studies. An increased Sin Nombre viral load is likely to produce a more severe clinical outcome (Xiao et al., 2006). HTNV RNA load in plasma in patients during the early stages of HFRS is associated with disease severity (Yi et al., 2013). Close correlation between viral load and disease severity were also found in cases of DOBV (Saksida et al., 2008).

It has been suggested that the cell permeability induced by hantavirus infection is associated with impaired barrier structure. An analysis of renal biopsy specimens from hantavirus-infected patients revealed that the expression and the localization of the tight junction protein ZO-1 were altered compared to renal biopsy specimens from non-infected individuals, that both tubular and glomerular cells were affected by the infection, and that the decrease in glomerular ZO-1 correlated with disease severity induced by glomerular dysfunction (Krautkrämer et al., 2011). It was reported that increased secreted vascular endothelial growth factor (VEGF) and concomitant decreased VE-cadherin were detected during the early stages in human primary lung endothelial cells infected by Andes virus (Shrivastava-Ranjan et al., 2010). The study also found that active virus replication could produce increased permeability and decreased the integrity of the endothelial cell barrier. Another study found that VEGF binding to VEGF receptor 2 (VEGFR2) may result in dissociation of VEGF-R2 from VE-cadherin, VE-cadherin activation, internalization, and degradation, that VEGF addition to ANDV- and HTNV-infected endothelial cells may induce the hyperphosphorylation of VEGFR2, and that concomitant with the VEGFR2 hyperphosphorylation, VE-cadherin may be internalized to intracellular vesicles within ANDV- or HTNV- infected endothelial cells (Gorbunova et al., 2010). Wang et al. (2012b) found the interaction between β3 integrin and VEGFR2 and the formation of a functional complex and that the signaling through this complex caused cytoskeletal reorganization, which was an important mechanisms underlying hyperpermeability. They also found that VEGF remarkably enhanced HTNV-directed permeability and the disruption of junctional organizations in an endothelial cell (EC) monolayer at 3 days postinfection.

### Immunity factor

**Figure 2:**
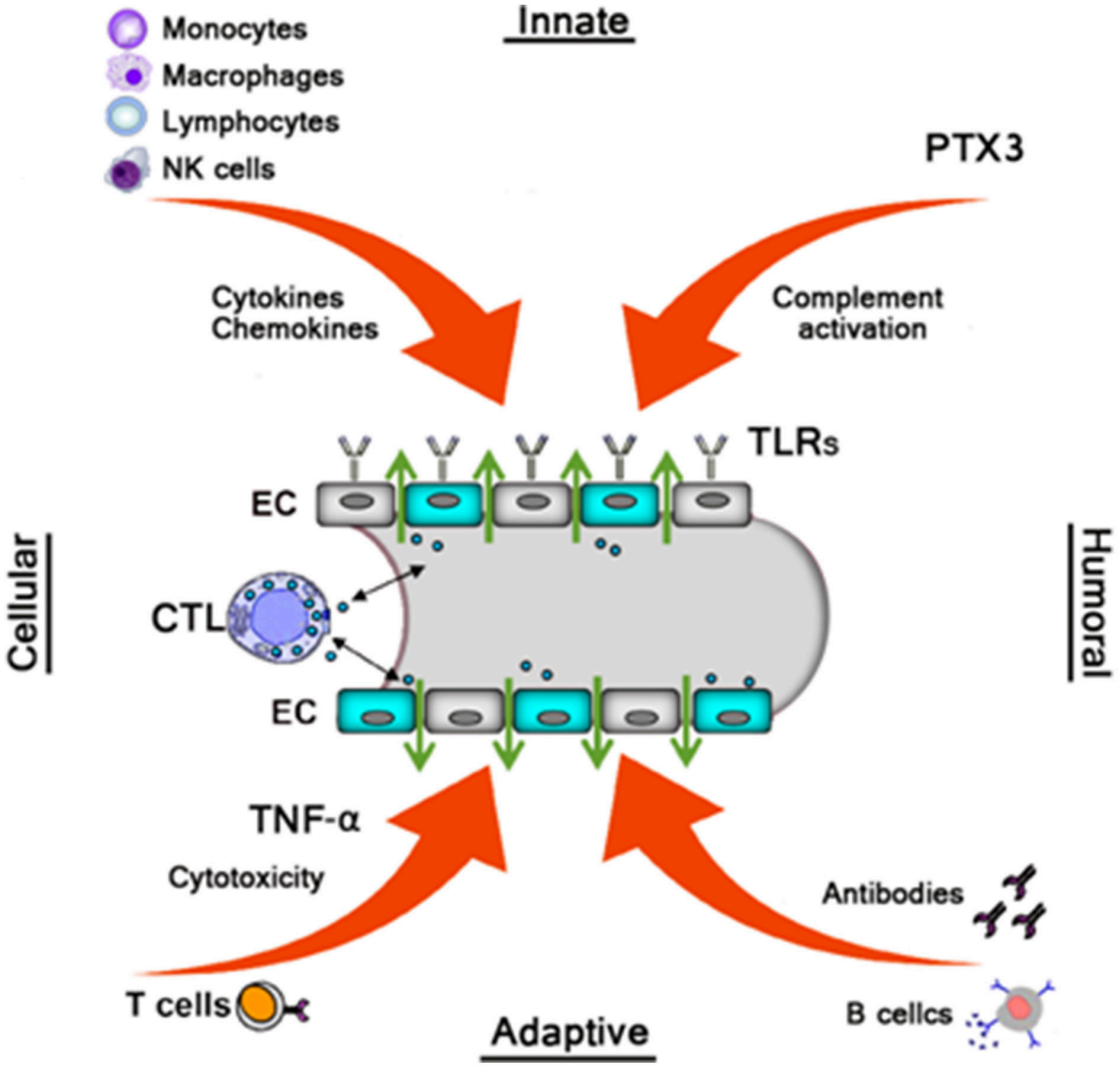
**Monocytes, macrophages, NK cells, and Lymphocytes produce various cytokines/chemokines which directly or indirectly increase vascular permeability**. The humoral pattern recognition receptor PTX3 and antibodies activate complement. Activated complement components induce cytoskeletal rearrangement in EC further increasing dysfunction of the EC barrier. TLRs recognize Hantavirus and mediate the innate response. Virus-infected ECs were cleared by virus-specific CTLs leading to vascular leakage. B cells produce several subclass antibodies, while only the neutralizing antibodies against G1 and G2 is beneficial to decrease the viruses, then decrease vascular leakage.

Similar to the effects of many other pathogenic viruses, HFRS is mainly medicated by the efforts of the immune system, both innate, and adaptive, to clear the infection. Therefore, it has been widely accepted that HFRS pathogenesis is largely immune mediated, including immune complexes, complement activation, T cell response, B cell response, and HTNV-induced cytokine production (Khaiboullina et al., 2005b; Easterbrook et al., 2007) (Figure 2).

#### Role of innate immune response

##### TLRs

Innate immunity works like a sentinel against microbial pathogen invasion. Innate immunity can be activated immediately following the recognition of diverse Pathogen-associated molecular patterns (PAMPs) by various Pattern-recognition receptors (PRRs). Among the different receptors that participate in the recognition of microbial invaders, Toll-like receptors (TLRs) play important roles in mediating the innate response (Akira et al., 2006). TLRs can produce effective immune responses and trigger the release of inflammatory cytokines and type I interferon for host defense (Beutler, 2009). Handke et al. (2009) found that HTNV may trigger TLR3-dependent innate immune response. In Jiang et al.'s study (2008), five TLRs (TLR2, TLR3, TLR4, TLR7, and TLR9) were detected in HTNV-infected vascular endothelial cells; however, only the expression of TLR4 was up-regulated. They also found that TLR4 may mediate the up-regulated expression of TNF-α, interferon-β (IFN-β) and IL-6 in HFRS. Zhang et al. (2014b) found that HTNV, a single-strand RNA virus, can act as a double-strand RNA to activate the TLR, retinoic acid inducible gene I (RIG-I) and MDA-signaling pathways, that NF-κB and IRF, activated through the downstream transcription factors of these pathways, bound directly to the CXCL's promoter, which then increased the expression of CXCL, and that RANTES and IP-10 have been detected altered after HTNV infection.

##### Cytokines/chemokines

The overproduction of inflammatory cytokines is commonly reported in subjects with HFRS and has given rise to the hypothesis that a so-called “cytokine storm” may play a pivotal role in the pathogenesis of the disease. Vaheri et al. (2013) found that these molecules were produced by various cells, such as macrophages, monocytes, and lymphocytes, in response to pro-inflammatory signals and participate in the regulation of inflammation. Wang et al. (2012a) reported that the serum concentrations of TNF-α, IL-6, IFN-γ, IL-8, IP-10, and RANTS (but not IL-4) were remarkably higher in HFRS patients, compared with controls, and that the highest concentrations were usually found during the febrile, hypotensive, and oliguric phases, particularly in severe and critical-type HFRS cases. Increased levels of cytokines and chemokines and an imbalance in their production contribute to increasing vascular endothelial permeability and a severe clinical course in patients with HFRS. Lohoff and Mark (2005) suggested that cytokines, such as TNF-α, IL-6 and IL-1, were mediators inducing fever, septic shock and acute phase protein production, and that TNF-α, IFN-β, and IL-6 were important cytokines which were responsible for the increased permeability of endothelial cells.

Overproduction of TNF-α may lead to severe systemic toxicity and has to do with the symptoms of HFRS. TNF-α treatment of endothelial cells *in vitro* may lead to increased endothelial cell monolayer permeability without visible cytopathic effect. In Linderholm et al. study (1996), the level of TNF-α was found to be increased in plasma during the acute stage of hantavirus infection. However, Khaiboullina et al. (2000) found that TNF-α level did not change in SNV-infected cells and that supernatants from SNV-infected human alveolar macrophages did not induce an obvious increase in endothelial monolayer permeability.

It was reported that the levels of VEGF were closely correlated to the progression of HFRS. Moreover, elevated levels of VEGF have correlation with the severity and degree of kidney damage (Li et al., 2013). The β3 integrins regulate vascular permeability through effects on VEGF (Dvorak, 2006). The α_v_β_3_ works coordinately with VEGF to direct endothelial cell migration during angiogenesis and to reseal the endothelium in response to damage (Byzova et al., 2000).

Type I IFNs (IFNα/β) are cytokines which are produced in response to viral infection and play an important role in regulating viral replication. *In vivo* and *in vitro* studies demonstrated that higher levels of IFNβ occurred in the serum of HFRS patients and in hantavirus-infected cell (Murzabaeva, 2002; Jiang et al., 2008). It was reported that serum level of IFN-γ was upregulated in HFRS cases when compared to healthy controls and the level of upregulation is dependent on the phase and severity of the disease (Khaiboullina et al., 2014). They also observed an association between the mild form of the disease and elevated serum levels of IFN-γ and IL-12. Collectively, these observations indicate that the administration of exogenous IFN-γ and IL-12 may provide antiviral benefits for the treatment of HFRS. Thus, regulating the early IFN response is necessary for hantaviruses to replicate in human endothelial cells and to be pathogenic.

The IFN-inducible MxA is considered to be important for control of infections with hantaviruses. MxA inhibits representative members of the Bunyaviridae family by interacting with an early step of virus replication. MxA protein can inhibit PUUV and Tula virus (TULV) replication in both viral protein and RNA accumulation in virus-infected cells (Kanerva et al., 1996). When constitutively expressed in stably transfected Vero cells, MxA prevented the accumulation of viral transcripts and proteins of Hantaan virus (Frese et al., 1996). It was suggested that MxA endogenously expressed in response to type I or type II IFNs does not play a pivotal role in the antiviral process against HTNV and that there is more than one mechanism by which cellular defense blocks hantavirus replication (Oelschlegel et al., 2007). Khaiboullina et al. (2005b) found in their study that MxA inhibited production of new infectious virus particles by interacting with the virus nucleocapsid protein.

Several other cytokines also have relationship with the symptoms of HFRS. IL-6, IL-10, VEGF, TNF, and cytotoxic T cell-mediated mechanisms are associated with the symptoms of HFRS (Hayasaka et al., 2007; Gavrilovskaya et al., 2008). Increased levels of IL-6, TNF, IL-1, IL-8, IFN-γ, IP-10, and CCL5 were found *in vivo* and *in vitro* (Saksida et al., 2011; Wang et al., 2012a). High plasma IL-6 levels are associate with severe renal failure and thrombocytopenia in PUUV-induced HFRS and can be looked as a marker of disease severity (Outinen et al., 2010). Increased levels of IL-10, INF-γ and TNF-α were observed in most HFRS patients infected by DOBV. Moreover, the concentrations in patients infected with DOBV are higher than in patients infected with PUUV on average (Saksida et al., 2011).

To sum up, inflammatory cytokines/chemokines produced by the antiviral innate response is like a double edged sword. On the one hand, they are helpful in eliminating viruses directly or indirectly by inducing innate effector functions and antigen presentation of viral epitopes to T cells. On the other hand, inflammatory cytokines/chemokines may facilitate immunopathological processes in virus-associated diseases.

##### Complement

It has been proposed that the complement system is associated with hantavirus-induced immunopathology. The soluble form of the terminal complement membrane attack complex, SC5b-9, can increase the permeability of cultured endothelial cells (Bossi et al., 2004). In the acute phase of PUUV infection, the complement system becomes activated and the severity of disease varies directly as the levels of complement activation (Sane et al., 2012). The acute phase protein pentraxin-related protein 3 (PTX3), which mediated the complement activation in the course of hantavirus infection, is increased during the acute phase of PUUV infection (Outinen et al., 2012).

##### NK cell

Natural killer cells also participate in the pathology and the capillary leak syndrome in HFRS (Björkström et al., 2011). Monocytes and Macrophages put up a bridge between innate and adaptive immunity. High expression of cytokines activating in the early phase of HFRS contribute to the immune-mediated pathogenesis (Mustonen et al., 2013).

#### ROLE of cellular immune response

##### B cell response

*IgM response*. IgM antibody is against all the three structural proteins of hantavirus. Shortly after the onset of the disease, activation of the humoral immune response will often result in formation of IgM. HFRS patients are characterized by high levels of hantavirus IgM detected simultaneously with the onset of clinical symptoms. Serum levels of IgM will reach the maximum 7–11 days after initial symptoms. In the convalescent phase of the HFRS, the levels of the IgM usually decline meanwhile the levels of IgG rise (Khaiboullina et al., 2005a). Therefore, it is often used as a diagnosis index for HFRS.

*IgG subclass antibodies*. The levels of IgG1 and IgG3, often increase with progression of the disease. However, IgG2 normally remains at more or less the same level in HFRS patients (Lundkvist et al., 1993). During the convalescent phase of HFRS, IgG3 against hantavirus N and G1 proteins often reach the highest peak and then decline gradually in the future decades. There is no direct relationship between the severity of the disease and levels of IgG subclasses. Recent years, there is too few further study about IgG antibodies and the knowledge about IgG antibodies is still limited.

*IgA response*. IgA plays an important role in mucosal immunity, and aerosol is an important way of the spread of hantavirus. Therefore, the presence of neutralizing IgA may play a significant role in the recovery from acute infection and in long-term immunity. The highest levels of anti-PUUV IgA can be detected in the sera of acute and early convalescent phase. With the progression of the disease, the levels of IgA will gradually decrease. Thus, IgA antibody is regarded as the marker of an early stage of infection (Nicacio et al., 2000). However, the mechanisms involved in IgA anti-viral protection remain unclear.

*IgE response*. High levels of hantavirus-specific and total IgE can be detected in serum during the acute phase of the disease. They usually gradually decline with the progression of the disease (Alexeyev et al., 1994). Though hantavirus infection significantly induced increased serum levels of IgE, the levels of virus specific IgE were not correlated with the severity of the disease (Alexeyev et al., 1994). Further investigations need to be focused on the function of IgE in the pathogenesis of HFRS.

*Neutralizing antibodies*. G1 and G2 glycoproteins are accepted as the major antigens involved in induction of neutralizing antibodies during hantavirus infection. It was demonstrated *in vitro* that Monoclonal antibodies (MAbs) directed against G1 and G2 displayed neutralizing activity, while the MAbs against N protein did not (Xu et al., 2002). Therefore, MAbs against G1 or G2 have potential possibility to be used as a powerful agent to neutralize virus in patients during early phases of HFRS.

#### T cell response

T cells have a two-sided role in viral infections. On the one hand, they are essential components of the host defense against intracellular pathogens, and on the other hand, they are often at least partly responsible for the organ damage and symptomatic disease caused by viruses. Both roles have been demonstrated in murine models, but data from primary human infections is scare and conflicting (Rouse and Sehrawat, 2010). Regulatory T cells contribute to increased amounts of TGF-β protein in the lungs during persistent SEOV infection. Therefore, regulatory T cells may contribute to subclinical pathologic observations in rats during SEOV infection (Easterbrook et al., 2007). In HCPS, hantavirus-specific T cell response positively correlated with disease severity. While in HFRS there are different results reported in different articles (Terajima and Ennis, 2011). Several studies have linked increased immune activity, both innate and adaptive, to a more severe disease (Outinen et al., 2012). However, whether this is because the strong immune response is harmful, or a severe infection induces a stronger immune is still unclear.

Cell-mediated immunity appears to play a crucial role in the immune pathogenesis of HFRS. Some kinds of immune cells, particularly CD8+ T cells, may also involve in the pathogenesis of Hantavirus infection. It has been found that infection with HTNV results in CTL responses to immunodominant regions on the NP (Wang et al., 2009b). Strong CD8+ T cell response was observed in PUUV infected patients (Lindgern et al., 2011). Levels of HTNV-specific CD8+ T lymphocytes in patients with HFRS were associated with different phases of HFRS. In the fever phase, it was significantly elevated. The levels of HTNV-specific CD8+ T lymphocytes in PBMC of patients with HFRS were negatively correlated with the levels of creatinine and blood urea nitrogen in plasma (Xie et al., 2013). In persistently infected mice, hantavirus strongly suppresses the production of N-specific CD8+ T cells throughout the course of infection (Taruishi et al., 2007). The CTL response was believed to play an important role against viral infection, but only a few HTNV epitopes recognized by the CTLs have been found. Three novel CD8+ CTL epitopes, N197-205 (RYRTAVCGL), N245-253 (KLLPDTAAV), and N258-266 (GPATNRDYL), were confirmed on the nucleocapsid protein. The epitopes were restricted by various HLA alleles including A11, A24, and B7. They were highly conserved among the reported hantanviruses, supporting their potential use in vaccine designs (Wang et al., 2011).

In addition, the importance of Treg cells in infections is well established, but in effect, all of the data on acute infections comes from reservoir hosts (Keynan et al., 2008). On one hand, Treg cells play a key role in maintaining host homeostasis by limiting destructive inflammatory reactions and thus decrease immunopathology during antiviral immune responses. On the other hand, suppression of the host antiviral immune response by Treg cells may induce viruses to persist in the host (Belkaid and Rouse, 2005). It was reported that the frequency of CD4+CD25^high^ cells was decreased and inversely correlated with disease severity in Chinese HFRS patients (Zhu et al., 2009). Treg cells have also been proved to contribute to the persistent asymptomatic carrier of hantaviruses in their rodent hosts, by suppressing immune responses (Easterbrook et al., 2007; Schountz et al., 2007). It was found that the best predictor of severe clinical course in HFRS was the FOXP3+ Treg cell response, suggesting that the role of Treg cells in acute human hantaviral infections may be deleterious (Koivula et al., 2014).

### Host genetic factors influence the clinical outcome

#### Interaction of hataviurs and host

Integrins are important for regulating vascular permeability and migrating of endothelial cells. It has been demonstrated that HFRS-causing hantaviruses, HTNV, SEOV and PUUV, gain cellular entry via specific α_v_β_3_ or α_IIb_β_3_ integrins (Gavrilovskaya et al., 1998; Mou et al., 2006). These integrins are heterodimeric receptors composed of α and β subunits which can mediate cell-to-cell adhesion and platelet aggregation. Endothelial cells and platelets are prominent regulators of vascular functions and integrins play key roles in barrier functions of these cells (Peters and Khan, 2002). Most interestingly, pathogenic and non-pathogenic hantaviruses use different integrin receptors. Human integrins α_IIa_β_3_ which isexpressedon platelets and α_v_β_3_ which is expressed on endothelial cells can mediate cellular entry of HFRS- and HCPS- causing hantaviruses (Gavrilovskaya et al., 2008). In contrast, non-pathogenic or low pathogenic prospect hill virus (PHV) or TULV were found to enter the cell via integrin α_5_β_1_ (Gavrilovskaya et al., 2002). It was found that HTNV-infected endothelial cells specifically direct the adherence of calcein-labeled platelets while cells comparably infected with nonpathogenic TULV failed to recruit platelets to the surface of endothelial cell (Gavrilovskaya et al., 2010).

Interaction of the host immune system and hantaviruses are of great importance for hantavirus infection. The pathogenesis of hantaviruses infection in humans is obviously affected by immunological factors.

#### Individual susceptibility to hantavirus

The severity of HFRS infected by HTNV is positively correlated to the age of the patients (Du et al., 2014b). It was reported that most deaths from the hantavirus disease NE occurred in older persons, especially the patients >70 years of age (Hjertqvist et al., 2010). In China, it was demonstrated that the case fatality rate was higher among individuals ≥50 years of age than that among the younger individuals (Klein et al., 2011).

It has been suggested that the frequency of infection is generally higher in male patients. The reported overall male-female ratio for NE cases was 1.52:1 in Sweden during 1997–2007 (Hjertqvist et al., 2010). The incidence rate of HFRS were significantly higher among male individuals than among female individuals for all ages in China during 2004–2008 (Klein et al., 2011). However, in northern Sweden, the same number of persons of either sex might be infected with PUUV (Ahlm et al., 1994). Fortunately, the clinical outcome seemed not worse in males. In China, it was illustrated that the case fatality rates of HFRS were significantly lower among male patients than among female patients 20–39 and ≥50 years of age (Klein et al., 2011). The expression of the supposed PRRs of hantavirus differs according to the sexes. Female patients infected with SEOV express higher levels of Toll-like receptor (TLR-7), RIG-I and IFN regulatory factor-7 (IRF-7) than male patients (Hannah et al., 2008).

In humans, the clinical procedure of hantavirus infections is affected by host genes (Vaheri et al., 2013). In China, HLA-DRB1^*^09 and HLA-B^*^46-DRB^*^09 haplotypes in patients with HTNV-induced HFRS were significantly more frequent than the controls (Wang et al., 2009a). In Finland, HLA alleles HLA-B8, HLA-C4A^*^Q0, and HLA-DRB1^*^0301 were associated with severe form of PUUV infection (Deter et al., 2008) while the individuals with HLA-B27 were often mild cases (Mustonen et al., 1996). In the United States, the individuals with HLA-B^*^3501 allele have a higher risk of severe SNV-induced HCPS (Terajima and Ennis, 2011). In Belgian patients, low TNF transcription (a polymorphism at position-238) was associated with severe clinical course (Maes et al., 2006).

## Treatment and prevention

### Antiviarl drugs

Several antiviral drugs, including IFN-α, steroids and cyclophosphamide, have been used in clinical for various effects. Ribavirin (1-beta-D-ribofuranosy 11,2,4-triazole- 3-carboxamide) has been used in the treatment of HCPS and HFRS (Huggins et al., 1991; Chapman et al., 1999). Clinical trials have shown that, if begun early during clinical course, ribavirin can be effective against hantaviruses causing HFRS (Moreli et al., 2014). It was also reported that after the application of ribavirin, the mortality of patients with HFRS showed a significant increase but that of patients with HCPS not (Moreli et al., 2014). However, another investigation demonstrated that ribavirin needed using in the oliguric phase to prevent mortality (Rusnak et al., 2009). Icatibant, a drug which blocks the binding of bradykinin, might be effective in large numbers of patients with PUUV and other hantavirus infection (Vaheri et al., 2014).

Favipiravir (T-705, 6-fluoro-3-hydroxy-2-pyrazinecarboxamide) was reported to have a high activity against a panel of Bunyaviruses (La crosse, Punta Toro, Rift Valley fever, sandfly fever viruses, SNV, Andes virus) by cytopathic effect and virus yield reduction (Gowen et al., 2007; Safronetz et al., 2013).

MAbs against HTNV have been developed. In China, a single-dose intravenous injection of a murine MAb against HTNV was developed and applied in healthy volunteers (Xu et al., 2009; Hall et al., 2010). It has been demonstrated that the anti-HTNV MAbs have anti-HTNV activity both *in vitro* and *in vivo* (Xu et al., 2002). Therefore, Mabs could be an effective candidate for the treatment of HFRS.

In addition, small molecule inhibitors of hantavirus infection were also studied. For example, inhibitors targeting α_v_β_3_ integrins may be used as antiviral agents (Hall et al., 2010). Other small-molecule inhibitors which target VEGFR2 and SFK(Src family kinases) signaling responses are able to inhibit ANDV-induced endothelial cell permeability (Gorbunova et al., 2011). However, the therapeutic potential of these compounds needs to be further studied and refined both *in vitro* and *in vivo*.

### Supportive measures

The earlier the detection, the admission to ICU and the supportive treatment, the greater the reduction of mortality rate (Huggins et al., 1991). Maintaining fluid and electrolyte balance is a crucial and fundamental treatment method. In addition to rectifying kidney dysfunction, blood pressure and oxygenation also need to be maintained according to the precise monitoring of invasive or non-invasive homodynamics. Platelet transfusions can be used in severe cases with thrombocytopenia and obvious bleedings. Symptomatic treatment is also necessary for patients with headache and back pains.

Continuous renal replacement therapy (CRRT) has become an important and widely used therapy for critical HFRS patients accompanied with mutiorgan injury, ARDS, pulmonary edema, fluid overload, severe electrolyte disturbances, cerebropathy, and AKI. Intermittent hemodialysis (IHD) is often chosen as the first choice by clinicians for AKI patients with stable hemodynamic status and less potentially fatal complications.

## Summary

HTNV is a major agent causing HFRS. The disease is obviously very complex. We think that the dysfunction of all the organs is mainly due to the capillary leakage and further studies should be emphasized on this aspect. Although the pathogenesis of HFRS is still unclear, our understanding on this disease has improved in recent years.

As the initial reason of the disease, the virus itself plays an important role in the course of pathological development. With the progress of the virus nucleic acid detection technology, it has become more easy and accessible to detect hantavirus RNA and structural protein in clinical cases. The role of virus in different clinical stages and whole disease process needs to be further studied.

It has been widely accepted that the immune response plays an important role in hantavirus pathogenesis. A number of studies have focused on adaptive immunity in the past. High levels of hantavirus specific antibodies and presence of IC in the blood were detected. More and more scientists have been paying attention to early activation of immune response to hantavirus antigens. Recently, lots of studies indicate that innate immunity stimulates adaptive immunity as well as innate immunity itself has antiviral functions. We believe that future efforts should be made to find the mechanism of the innate immunity in the pathogenesis of the disease.

Strong antiviral treatment has improved the prognosis of many kinds of viral diseases. Therefore, a new potent antiviral drugs against hantavirus should be developed. It should be emphasized that more and more studies are now focusing on the pathogenesis of increased capillary leakage. This research direction may provide a great inspiration for the searches on effective therapies and exploring potential therapeutic targets. Further important questions mainly refer to the identification of risk factors, preventive measures and vaccination. Answers had better be found through close cooperation from scientists of various fields.

## Author contributions

Wrote the paper: HJ, HD, LW. Revised the manuscript: PW, XB.

## Funding

This work was supported by the National Natural Science Foundation of China (No.81373118), the National Basic Research Programme of China (973 Programme No.2012CB518905), and the Substantial Development Program of Technology Innovation and Clinical Research in Tangdu Hospital (2013-4241370U2).

### Conflict of interest statement

The authors declare that the research was conducted in the absence of any commercial or financial relationships that could be construed as a potential conflict of interest.
