# Corrigendum: Hemorrhagic Fever with Renal Syndrome: Pathogenesis and Clinical Picture

**DOI:** 10.3389/fcimb.2016.00178

**Published:** 2016-12-08

**Authors:** Hong Jiang, Hong Du, Li M. Wang, Ping Z. Wang, Xue F. Bai

**Affiliations:** ^1^Center for Infectious Diseases, Tangdu Hospital, Fourth Military Medical UniversityXi'an, China; ^2^Department of Microbiology, School of Basic Medicine, Fourth Military Medical UniversityXi'an, China

**Keywords:** hantavirus, hemorrhagic fever with renal syndrome, Bunyavirus, hantaan virus, pathogenesis

Due to an oversight the authors did not cite the original source for **Figures 1, 2**. **Figure 1** was adapted from Figure 5 of Schönrich et al. (2008). The revised figure caption should read:

**Left side:** Normal endothelial cells (EC), no vascular leakage occurs. **Right side:** EC were infected with hantaviruses. ZO-1, VEGFR2, VE-cadherin on EC were altered. High hantavirus RNA load result in severe vascular leakage. Virus-infected ECs be cleared by virus-specific CTLs leading to vascular damage. Owing to acute thrombocytopenia, there are not sufficient platelets available to repair “holes” in the EC barrier, resulting in vascular leakage. In addition, cytokines produced during the innate response against pathogenic hantaviruses like TNF-α could enhance vascular permeability. Adapted from Schönrich et al. (2008).

**Figure 2** was adapted from Figure 2 of Schönrich et al. (2015). The revised figure caption should read:

**Monocytes, macrophages, NK cells, and Lymphocytes produce various cytokines/chemokines which directly or indirectly increase vascular permeability**. The humoral pattern recognition receptor PTX3 and antibodies activate complement. Activated complement components induce cytoskeletal rearrangement in EC further increasing dysfunction of the EC barrier. TLRs recognize Hantavirus and mediate the innate response. Virus-infected ECs were cleared by virus-specific CTLs leading to vascular leakage. B cells produce several subclass antibodies, while only the neutralizing antibodies against G1 and G2 is beneficial to decrease the viruses, then decrease vascular leakage. Adapted from Schönrich et al. (2015).

This does not affect the scientific conclusions of this article in any way. The authors apologize for this oversight.

## Conflict of interest statement

The authors declare that the research was conducted in the absence of any commercial or financial relationships that could be construed as a potential conflict of interest.
